# Structured Illumination Microscopy and a Quantitative Image Analysis for the Detection of Positive Margins in a Pre-Clinical Genetically Engineered Mouse Model of Sarcoma

**DOI:** 10.1371/journal.pone.0147006

**Published:** 2016-01-22

**Authors:** Henry L. Fu, Jenna L. Mueller, Melodi J. Whitley, Diana M. Cardona, Rebecca M. Willett, David G. Kirsch, J. Quincy Brown, Nimmi Ramanujam

**Affiliations:** 1 Department of Biomedical Engineering, Duke University, Durham, North Carolina, United States of America; 2 Department of Pharmacology & Cancer Biology, Duke University School of Medicine, Durham, North Carolina, United States of America; 3 Department of Pathology, Duke University School of Medicine, Durham, North Carolina, United States of America; 4 Department of Electrical and Computer Engineering, University of Wisconsin—Madison, Madison, Wisconsin, United States of America; 5 Department of Radiation Oncology, Duke University School of Medicine, Durham, North Carolina, United States of America; 6 Department of Biomedical Engineering, Tulane University, New Orleans, Louisiana, United States of America; Van Andel Research Institute, UNITED STATES

## Abstract

Intraoperative assessment of surgical margins is critical to ensuring residual tumor does not remain in a patient. Previously, we developed a fluorescence structured illumination microscope (SIM) system with a single-shot field of view (FOV) of 2.1×1.6 mm (3.4 mm^2^) and sub-cellular resolution (4.4 μm). The goal of this study was to test the utility of this technology for the detection of residual disease in a genetically engineered mouse model of sarcoma. Primary soft tissue sarcomas were generated in the hindlimb and after the tumor was surgically removed, the relevant margin was stained with acridine orange (AO), a vital stain that brightly stains cell nuclei and fibrous tissues. The tissues were imaged with the SIM system with the primary goal of visualizing fluorescent features from tumor nuclei. Given the heterogeneity of the background tissue (presence of adipose tissue and muscle), an algorithm known as maximally stable extremal regions (MSER) was optimized and applied to the images to specifically segment nuclear features. A logistic regression model was used to classify a tissue site as positive or negative by calculating area fraction and shape of the segmented features that were present and the resulting receiver operator curve (ROC) was generated by varying the probability threshold. Based on the ROC curves, the model was able to classify tumor and normal tissue with 77% sensitivity and 81% specificity (Youden’s index). For an unbiased measure of the model performance, it was applied to a separate validation dataset that resulted in 73% sensitivity and 80% specificity. When this approach was applied to representative whole margins, for a tumor probability threshold of 50%, only 1.2% of all regions from the negative margin exceeded this threshold, while over 14.8% of all regions from the positive margin exceeded this threshold.

## Introduction

Cancer is a complex and devastating disease that is continually among the top causes of death worldwide. For solid tumors, surgical intervention is commonly used to de-bulk the mass [1]. One example is the resection of extremity soft tissue sarcomas where surgeons seek to remove the entire tumor while preserving limb function. Assessment of the quality of the excision is performed by a pathologist after surgery. Positive margin status, which is indicated by the presence of tumor cells at the edge of the resected specimen, has been reported to correlate with local recurrence [2]. Without adjuvant radiation therapy, microscopic disease left at the surgical site causes local recurrence in up to 31% of sarcoma patients [2, 3]. Another example is breast cancer, the most commonly occurring cancer type in women [4]. Breast conserving surgery (BCS) is a surgical procedure where the surgeon attempts to remove only cancerous tissues as opposed to the more radical mastectomy operation, where the entire breast is removed [5]. It is estimated that 59% of patients with breast cancer undergo BCS [6] Unfortunately, 20–70% of these patients must undergo repeat surgery due to incomplete removal of residual disease during the initial operation [7–13]. Intraoperatively, surgeons typically have limited feedback on whether disease has been completely removed at the time of surgery.

Currently there are two clinically used intraoperative techniques that are employed to assist surgeons in the operating room. The first technique is frozen section, where the specimen is immediately flash-frozen and sectioned [14–17]. These tissue sections are stained with hematoxylin and eosin (H&E) and then sent to a pathologist for an immediate diagnosis that is relayed back to the surgeon. The second technique is imprint or touch-prep cytology [18, 19]. Once the specimen has been removed, the margin is touched to a glass slide, which is inspected by a trained cytologist. Both of these techniques have demonstrated success in reducing re-excision rates by up to 34% [20]. Unfortunately, both of these techniques are resource-intensive and require highly trained personnel, limiting their widespread availability.

Fluorescence microscopy, combined with vital stains has the ability to visualize microscopic features in tissues and is on par with reported times used by pathologists to examine intraoperative frozen sections [21]. For example, a nuclear contrast agent, such as Acridine Orange (AO), can be topically applied to the relevant margin, so minimal tissue preparation would be required [21–29]. The value of fluorescence microscopy, unlike conventional pathology, lies in the possibility of being able to directly image fresh tissue specimens immediately after removal from the patient. However, the approach is not without drawbacks, as the resected tumor mass from a patient is optically thick and contributes to the scattering of light, thus creating large amount of unwanted background fluorescence. Additionally, the contrast agent can diffuse into deeper layers of thicker tissue specimens thus generating even more fluorescence signal from outside the focal plane. This source of background fluorescence severely degrades contrast.

To address this issue, we have previously developed a fluorescence microscope that employs a specialized technique called structured illumination microscopy (SIM) to reject background fluorescence [30]. Rather than simply illuminating the sample with a uniform pattern, a spatially varying sinusoid pattern is projected onto the sample. This specific illumination pattern only modulates the fluorescence emission of objects at the focal plane, while any scattered fluorescence or fluorescence originating from deeper layers is not modulated, and thus rejected by a simple nonlinear decoding algorithm [30]. The SIM system our group has developed was specifically designed and optimized for imaging physically thick tissues stained with AO, with varying optical sectioning thicknesses achieved by varying the normalized pattern spatial frequencies of the illumination (in this specific implementation the optical section thickness was 128 μm) [31].

The present study builds on our previous work where SIM was used for the detection of positive tumor margins in a genetically engineered mouse model of sarcoma [31]. While fresh, intact tissue was only imaged from one mouse in our previous work, this new study presents tissue images collected from a larger cohort of 23 tumor-bearing mice. Additionally, an advanced image processing algorithm known as maximally stable extremal regions (MSER) was optimized and used to segment nuclear features in images acquired with SIM and a logistic regression model was used for the purposes of classification [32]. Collecting data from a larger mouse dataset was critical in optimizing MSER segmentation and the classification algorithm. Additionally, the larger dataset enabled us to determine the sensitivity and specificity for positive margin detection using this combination of methodologies. The results of this study demonstrate that SIM, in conjunction with MSER, effectively isolates nuclear features in heterogeneous thick tissues that are significantly more prevalent in cancer-positive compared to cancer-negative tumor margins.

## Methods

### Ethics statement

This study was carried out in strict accordance with the recommendations in the Guide for the Care and Use of Laboratory Animals of the National Institutes of Health. The protocol was approved by the Duke University Institutional Animal Care and Use Committee (Protocol Number: A109-13-04). All surgery was performed under isoflurane gas anesthesia, and all efforts were made to minimize suffering.

### SIM system

The SIM system used in this study was fully characterized and described in detail in a previous publication [31]. The system specifications (excitation wavelength, filters, and dichroic mirror) were optimized to image acridine orange (AO), which in thick tissues. Cell nuclei, skeletal muscle, and collagenous stroma have all been shown to generate fluorescence after non-specific staining and uptake of AO.

Briefly, the system consists of a broadband light source (Fianium SC400) with an excitation filter at 480 nm (FWHM = 20 nm). The light is projected onto a LCoS SLM display chip (Holoeye LCR-720) to generate the 2-D sinusoid illumination pattern. A 4x objective (Nikon E Plan Fluor, NA = 0.1) is used for epi-fluorescence illumination with a resolution of at least 4.4 μm and field-of-view of 2.1x1.6 mm. The fluorescence emission light is captured using a scientific grade CCD (LaVision Imager 3 QE, 12-bit, 1376x1040, 6.45 μm square pixels). The results of the study showed that an illumination frequency of 31.7 μm^-1^ provides the optimal tradeoff between optical section thickness and signal-to-noise ratio (SNR). At this specific illumination frequency, the measured optical section thickness was 128 μm.

### Margin imaging protocol

To simulate clinical tumor growth and disease progression, a genetically engineered mouse model of sarcoma was selected as a primary test bed for margin classification [33]. All animal experiments were approved by the Duke University Institutional Animal Care and Use Committee (IACUC). Mice with conditional mutations in oncogenic KRAS or BRAF and P53 were injected intramuscularly in their hindlimb with an adenovirus expressing Cre recombinase to induce primary sarcomas [34].

After injection of the adenovirus, the sarcoma was allowed to develop and grow to 500–700 mm^3^ (60–90 days post injection) and then surgically removed from the animal. The mouse was anesthetized with isofluorane for the duration of the surgical procedure. To limit pain, the mice were administered a subcutaneous bolus of buprenorphine and ketoprofen before incision. During the procedure, the entire leg, including the tumor mass, was amputated to expose the relevant margin. The excised tumor margin was immediately prepared for imaging by topically applying a 0.01% aqueous solution of AO and then rinsed with 0.1M phosphate-buffered solution (PBS). To obtain the highest quality images, a cover glass was placed on top of the tumor margin to create a flat focal plane for imaging.

**Fig 1:**
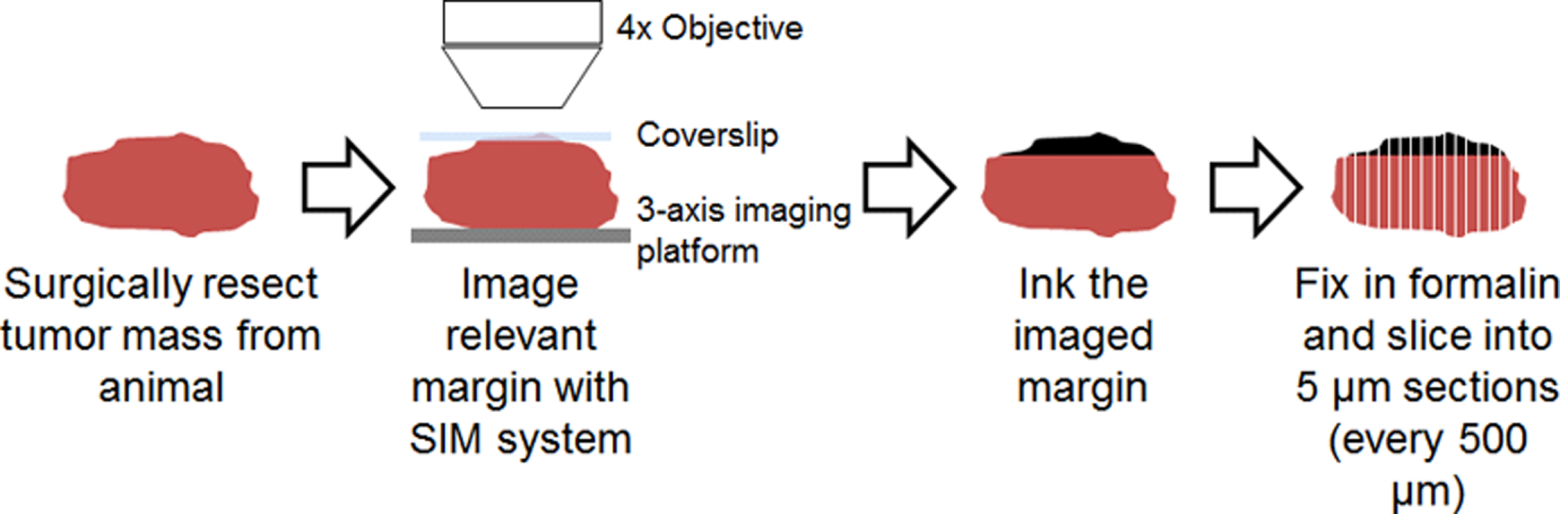
Specimen imaging protocol. Flowchart demonstrating the imaging protocol immediately after the tumor margin was removed from the transgenic sarcoma model. After the procedure above was completed, the tissue sections were inked to identify the area imaged, stained with H&E and submitted to pathology.

A brief overview of the imaging procedure is shown in Fig 1. The sample was placed on a 3-axis translation stage under the imaging objective for the purposes of focusing and lateral translation. Due to the size of the margins (~3x5 mm), multiple images were acquired to cover a single margin. Beginning at the first image location, the sample was translated to each subsequent image location using a micrometer to ensure the sample was moved an equal distance between image sites. A small amount of overlap between adjacent images was allowed to ensure the entire margin was imaged. The sample was moved 1.3 mm in the x-axis and 1.8 mm in the y-axis, with a typical margin fully imaged using 4–6 frames.

To correlate the imaging results obtained with the SIM system to pathology, tattoo ink was painted over the region of the tissue that was imaged, which allowed the pathologist to identify the corresponding margin after fixing and slicing the tissue. After applying ink, the tissue was immediately frozen in optimal cutting temperature gel (OCT) using liquid nitrogen. A microtome was used to slice 5 μm thick tissue sections which were perpendicular to the inked imaged surface and spaced 500 μm apart. The tissue sections were then fixed in formalin and then stained with H&E. A pathologist reviewed all slides from a given margin to classify it either as a positive or negative for tumor cells. The criterion used to determine a positive margin was to identify the presence of any tumor cells directly in contact with ink. The exact number of H&E slides varied based on the physical size of the margin (average of 21 slides per margin).

### Image processing algorithm

The images obtains of the sarcoma margins exhibit clear attributes, such as nuclear density and shape, that can be used to differentiate positive from negative margins. A quantitative image analysis method was developed to automate margin classification using SIM data. This approach has three main steps: (1) low-level image feature extraction, (2) calculation of nuclear density and shape statistics from low-level features, and (3) logistic regression on the output of the nuclear statistics to classify the margin.

#### Low-level image feature extraction

The SIM margin images are generally composed of a heterogeneous mix of tumor, muscle, adipose, and other various tissues. A technique called maximally stable extremal regions (MSER) are often used in image analysis for “blob detection”, or finding groups of bright pixels superimposed on a varying background intensities, and hence are an appropriate choice for nucleus segmentation[32]. MSER utilizes basic intensity thresholding; however, no global or optimal threshold is sought, rather all thresholds are tested and the stability of the isolated connected components (i.e. nuclei) are evaluated. More specifically, all possible thresholds are applied to an image and all sets of connected components (adjacent pixels with intensity values that exceed the current threshold) that are isolated with each threshold are stored. This yields a data structure in which the area of each connected component is stored as a function of the intensity threshold. Finally, the intensity threshold that corresponds to a local minimum in the calculated size variation function (shown in Eq.1 below) for each connected component is selected as a threshold producing MSER.

#### MSER parameter tuning procedure

In order to apply MSER specifically to our images, five parameters associated with MSER, needed to be tuned and selected. The descriptions of all variables are provided in Table 1. The two most straightforward parameters are MinArea and MaxArea, which are related to the expected size of the connected components (i.e. nuclei). These parameters were selected based on the biologically expected range of nuclear diameters. Specifically, MaxArea was set to a 15 pixel area, which corresponds to greater than 20 μm in diameter (assuming circular area), the largest expected nuclear size for the sarcoma mouse model [35]. MinArea was set to 3 pixels, which correspond to less than 5 μm in diameter, which is the smallest expected nuclear size for the sarcoma model [35]. The next set of parameters was related to the intensity thresholds and included MaxVariation, MinDiversity, and Delta. These intensity parameters were systematically tuned through the range of values seen in Table 1 on 30 representative images containing distinct tissue types, 10 tumor, 10 muscle, and 10 adipose. These specific tissue types were chosen due to their common occurrence in the original 23 mice dataset.

**Table 1:** Parameters used in MSER algorithm.

Parameter	Description	Range tested
MinArea	Minimum allowed size of connected component region. Selected based on minimum expect nuclei size.	[3] (pixels)
MaxArea	Maximum allowed size of connected component region. Selected based on maximum expect nuclei size.	[15] (pixels)
MaxVariation	Maximum intensity variation allowed within in a region	[0 50] (intensity value, 8-bit depth)
MinDiversity	When the relative variation of two nested regions is < MinDiversity, then only the most stable region is selected	[0 1] (normalized units)
Delta	Related to the intensity stability of a region of connected components. The stability of a region is the relative variation of the region area when the intensity is changed of ±0.5*Delta.	[2 50] (intensity value, 8-bit depth)

The MSER parameters that are related to intensity measured at each pixel. A short description of the function of each parameter and range of values used during the optimization procedure is given.

**Fig 2:**
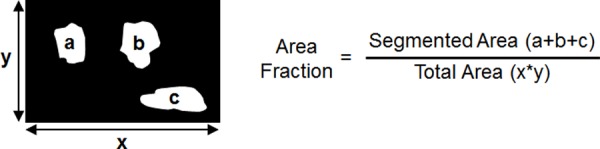
MSER terminology explanation. Methodology for calculating area fraction of a MSER segmented image. Area fraction was the metric used to quantify the performance of the MSER algorithm.

Each image was a region of interest cropped from a margin to ensure that only the specified tissue type was present. The images were processed multiple times using the MSER algorithm while slightly varying the parameters for each trial. The change in area fraction, which is the segmented area divided by total image area (shown in Fig 2), was calculated at each trial to quantify the segmentation performance. Area fraction served as a comprehensive quantitative value as it was dependent on both density and size of segmented areas. The goal of the tuning procedure was to select parameter values that yielded the least variation in area fraction. In addition, the segmented images using the final selected parameters were qualitatively inspected to ensure that nuclei were accurately segmented based on physiological expectation.

#### High-level image statistics computed from low-level features

We developed and optimized a predictive model to distinguish between images of distinct tissue types. The images used to tune the MSER parameters (10 tumor, 10 muscle, and 10 adipose) were used as a training dataset for constructing the site level-predictive model. This dataset is available for download at the following link: http://dukespace.lib.duke.edu/dspace/handle/10161/10892. Each image was a 350x300 pixel region (525x450 μm) of interest (ROI) from the original dataset of 23 margins. This ROI size was chosen because it was roughly equivalent to the FOV of a 10x objective, a typical magnification used by pathologists when studying a suspicious region.

**Fig 3:**
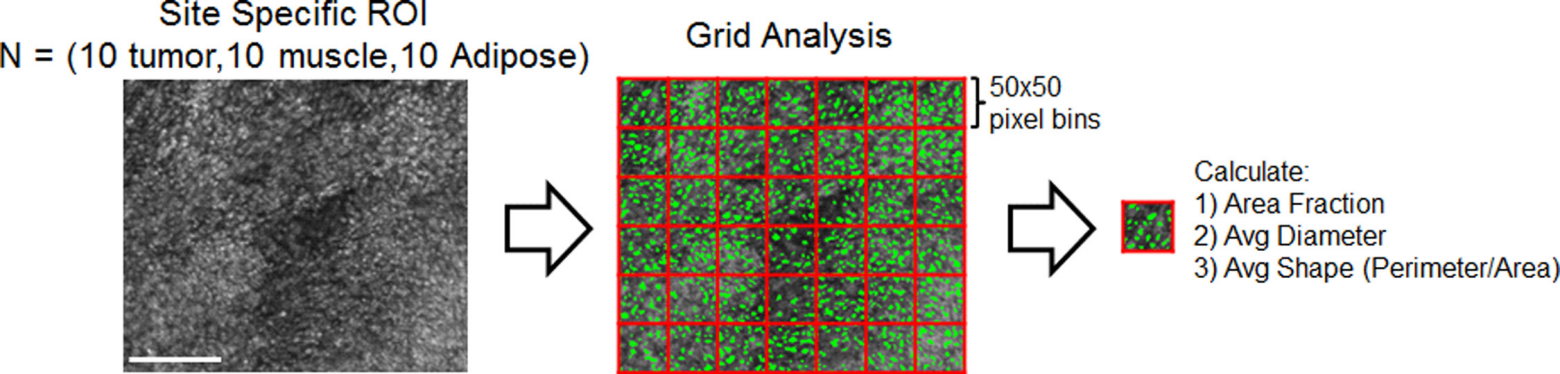
Site-level analysis. Flow chart which demonstrates the quantitative analysis carried out on the site specific ROIs. The purpose of this analysis was to develop a predictive model to differentiate tumor tissue from muscle and adipose. A total of 30 images were used as a training dataset to develop the model. Each image was further divided into smaller bins to ensure very localized disease could be detected. Finally, area fraction, average diameter, and average shape were calculated for each bin and used as input variables for a logistic regression model. Scale bar is 200 μm.

One additional step was taken to further divide each image into smaller elements; each image was broken into 42 evenly spaced 50x50 pixel bins. This procedure is shown visually in Fig 3. The rationale for this step was if classification was carried out on the 350x300 pixel ROIs, it is likely that small focal areas of tumor would not be detected. A bin size of 50x50 pixels was chosen because this corresponded to the diameter of a single skeletal muscle fiber or a single adipose cell. After this step, the true number of measurements in the training dataset was N = 1260 bins (30 images with 42 bins per image) with N = 420 tumor bins, N = 420 muscle bins, and N = 420 adipose. Using the segmentation output from the MSER algorithm, three different variables were calculated for each bin, area fraction, average diameter, and average shape (perimeter/area).

#### Logistic regression applied to high-level image statistics

Once the segmented area fraction, average diameter, and average shape were quantified for each bin, the goal was to construct a predictive classification model to differentiate tumor bins from normal bins. While the training dataset contained three distinct tissue types, a binary logistic regression model was chosen for the classification algorithm. To reduce the three group training dataset to a binary dataset, the adipose and muscle tissue were classified together as negative tissue sites. The three variables used as inputs into the logistic regression model were the area fraction, average diameter, and average shape for each 50x50 pixel bin.

## Results

In total, tumors from 23 mice were imaged following the procedure described in the Methods section. In addition to imaging with the SIM system, each margin was also diagnosed by a pathologist (DMC) with expertise in connective tissue pathology. From the dataset of 23 total margins, 15 margins were positive and 8 margins were negative. It should be noted that in a seven mice, the tattoo ink was lost during the tissue processing and the pathologist was unable to visually detect the ink in the H&E slides. However, these margins were still diagnosed based on the following alternate criteria. A margin was diagnosed as negative if a tumor was not circumferentially present. However, if tumor cells were visible at the edge of the tissue sections, the margin was diagnosed as positive. The final breakdown of mice was 15 positive margins (12 with ink on H&E, 3 without ink on H&E) and 8 negative margins (4 with ink on H&E, 4 without ink on H&E).

### MSER optimization results

**Fig 4:**
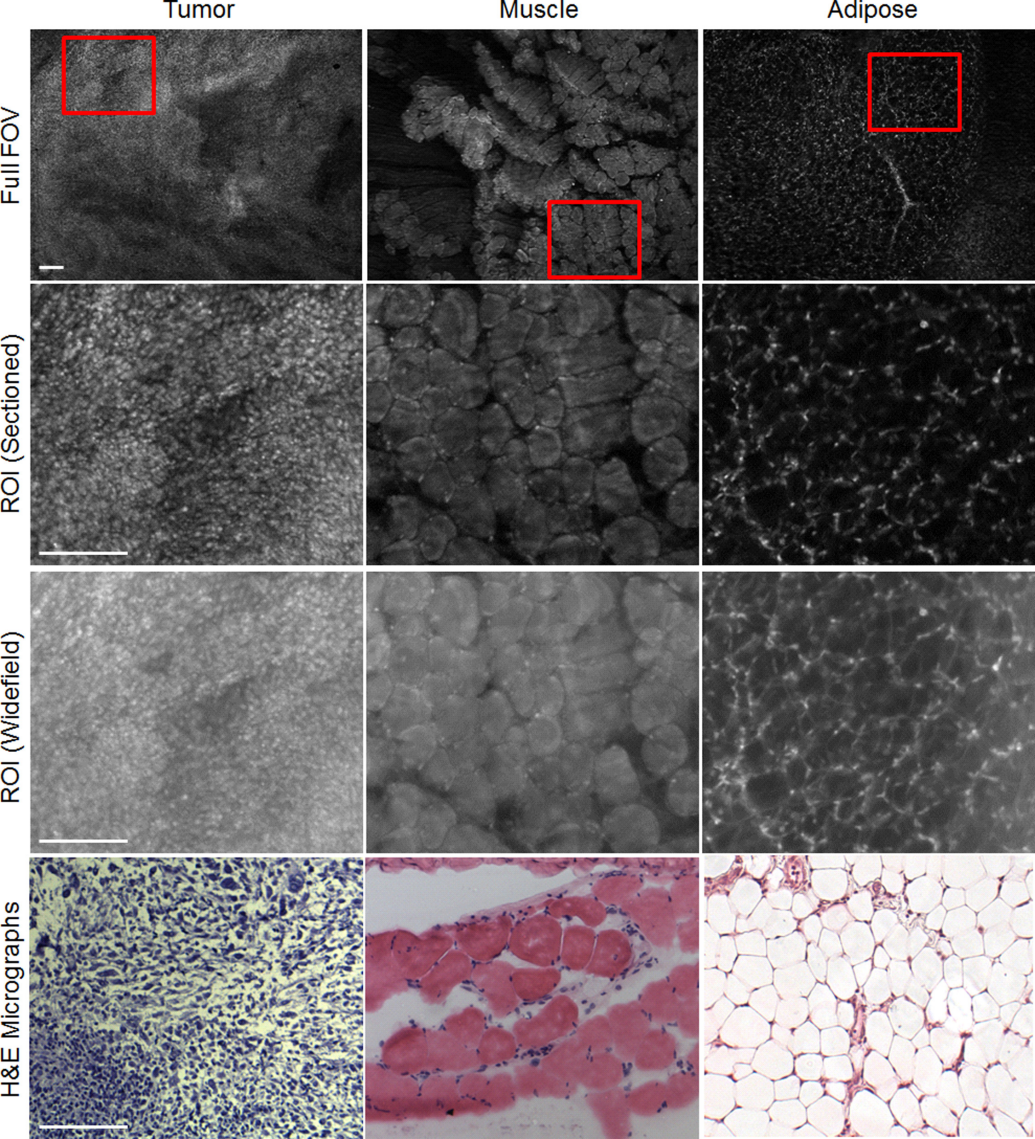
Site-level images. Representative SIM images of three different tissue types commonly found in margin images: tumor, muscle, and adipose tissues. For the smaller ROIs, both the sectioned (structured illumination) and widefield (standard illumination) images are shown to demonstrate the enhanced contrast that SIM provides. Examples of H&E micrographs are also shown, but it should be noted the H&E is not taken from the exact site of the corresponding SIM images because specific site level fiducial markers were not used in this study. Scale bars are 200 μm.

The first step in the image analysis was to optimize MSER for the images acquired with the SIM system to segment and quantify image features. However, as mentioned in the methods section, three specific parameters (MaxVariation, MinDiversity and Delta) were tuned to ensure the algorithm was accurately segmenting these features. A dataset consisting of 10 tumor, 10 skeletal muscle, and 10 adipose images were used to optimize the parameters. One representative image of each tissue type is shown in Fig 4. Pure sarcoma tumor tissue consists mostly of tumor cells with very little organizational structure. However, the density of the tumor tissue can vary, so tumor nuclei can potentially appear with a low background (lower density, loosely packed cells) or high background (higher density, heavily packed cells). Muscle nuclei are located on the periphery of muscle fiber bundles, which are also brightly stained with AO. As a result, nuclei associated with muscle typically appear on a bright background. In adipose tissue, the nuclei are located on the periphery of an adipocyte, but the lipid droplet that occupies the majority of adipocyte does not emit fluorescence from AO. The adipose nuclei appear on a mostly dark background, with fine streaks of background fluorescence from connective tissue.

**Fig 5:**
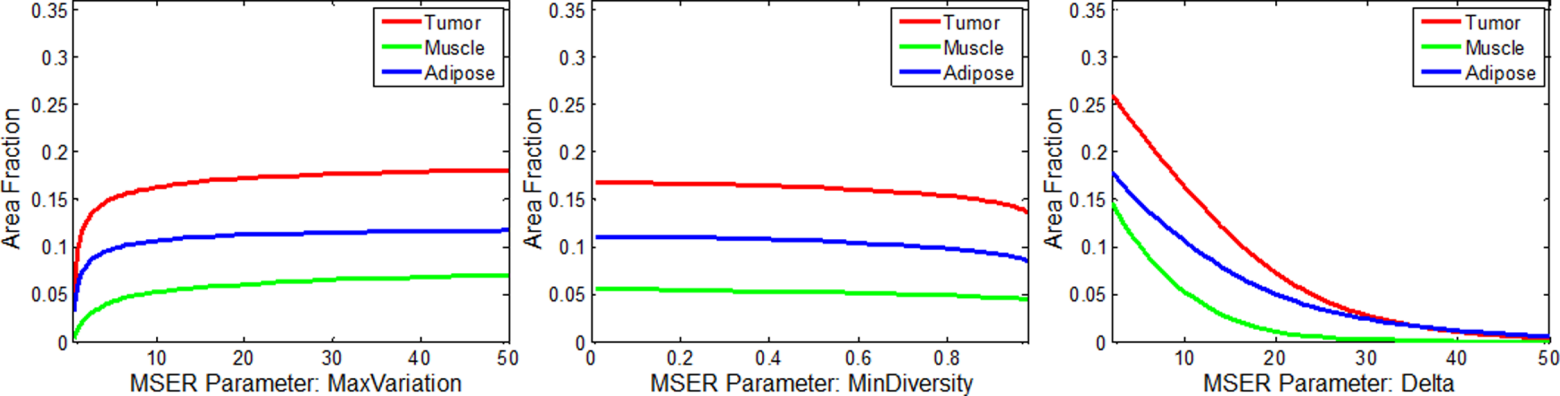
MSER parameter optimization. Plots that demonstrate the effect of three different MSER parameters on segmented area fraction for tumor, muscle, and adipose images (shown in previous figure). The parameter Delta shows the most impact on area fraction, while MaxVariation and MinDiversity have a minimal effect.

The results from the MSER parameter tuning procedure are shown in Fig 5 where the corresponding area fraction (averaged over all 10 images of each respective tissue type) is plotted for the case where one parameter is varied while the other two are held constant. The MSER output appears to be insensitive to MaxVariation and MinDiversity, since varying these parameters has minimal effect on area fraction. For the MaxVariation parameter, area fraction reaches a plateau for values greater than 10. The area fraction only increased by about 10% after exceeding a MaxVariation of 10. In MinDiversity, almost the entire range is a plateau and the area fraction shows very little variability regardless of the setting (15%). In contrast, varying Delta had the most significant impact on area fraction with the only visible plateau occurring when all area fractions converge to 0. Ultimately, a MaxVariation of 10 and MinDiversity of 0.5 were selected as these were within their respective plateau ranges. However, selecting Delta was not as straightforward.

**Fig 6:**
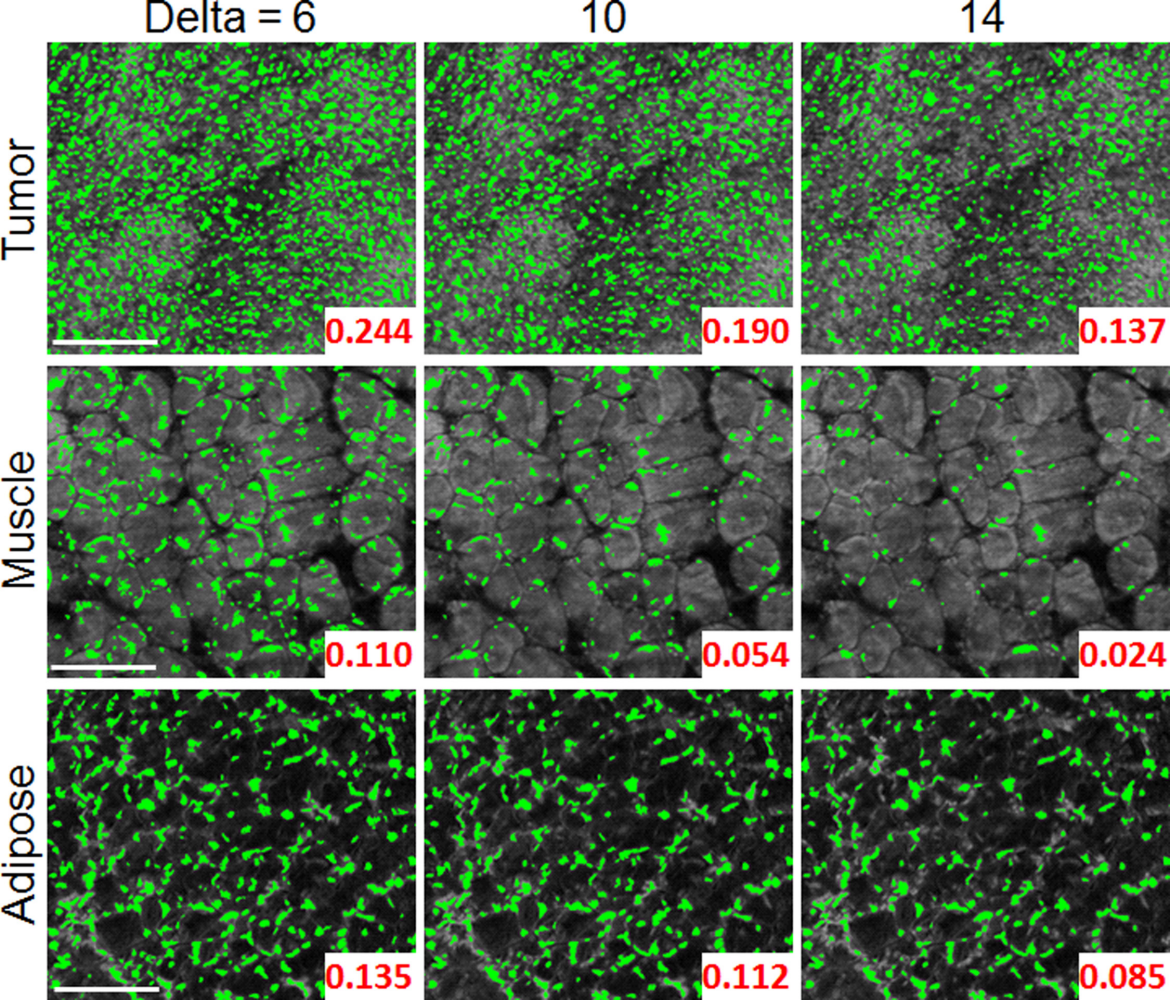
Tuning of Delta in MSER optimization. Series of images which demonstrate the impact of changing Delta on MSER feature segmentation. The calculated area fraction for each image is displayed on the bottom right. As shown in the previous plot, the segmented area fraction varies greatly as Delta changes. Scale are 200 μm.

To determine the best Delta value, the segmented images for the three representative images for Delta values between 2 to 14, shown in Fig 6, were visually inspected. Images with Delta > 14 were not included because the area fraction value of the tumor began to converge with the adipose, which does not match physiological expectations. In the set of images shown in Fig 6, the original SIM images have been overlaid with false coloring to represent the segmented areas identified with MSER. The numbers displayed in the bottom right is the corresponding area fraction of the MSER-segmented regions over total image area. As expected, the tumor area fraction is noticeably higher.

Based on qualitative inspection, Delta = 10 yielded the most appropriate and physiologically accurate segmentation results across representative images. At a Delta value of 6, the images appear over-segmented. This was particularly obvious when inspecting the representative skeletal muscle images where extensive areas inside the fiber bundles (which contain no nuclei) were segmented. For Delta = 14, the images were under-segmented, meaning some potential nuclei were not identified. Specifically, the adipose image for this Delta value clearly displays nuclei that are not highlighted. From inspection of these images, a qualitative justification for the selection of Delta was provided based on physiological expectation. However, the final decision was made with the support of quantitative analysis, which is shown in the following section.

**Fig 7:**
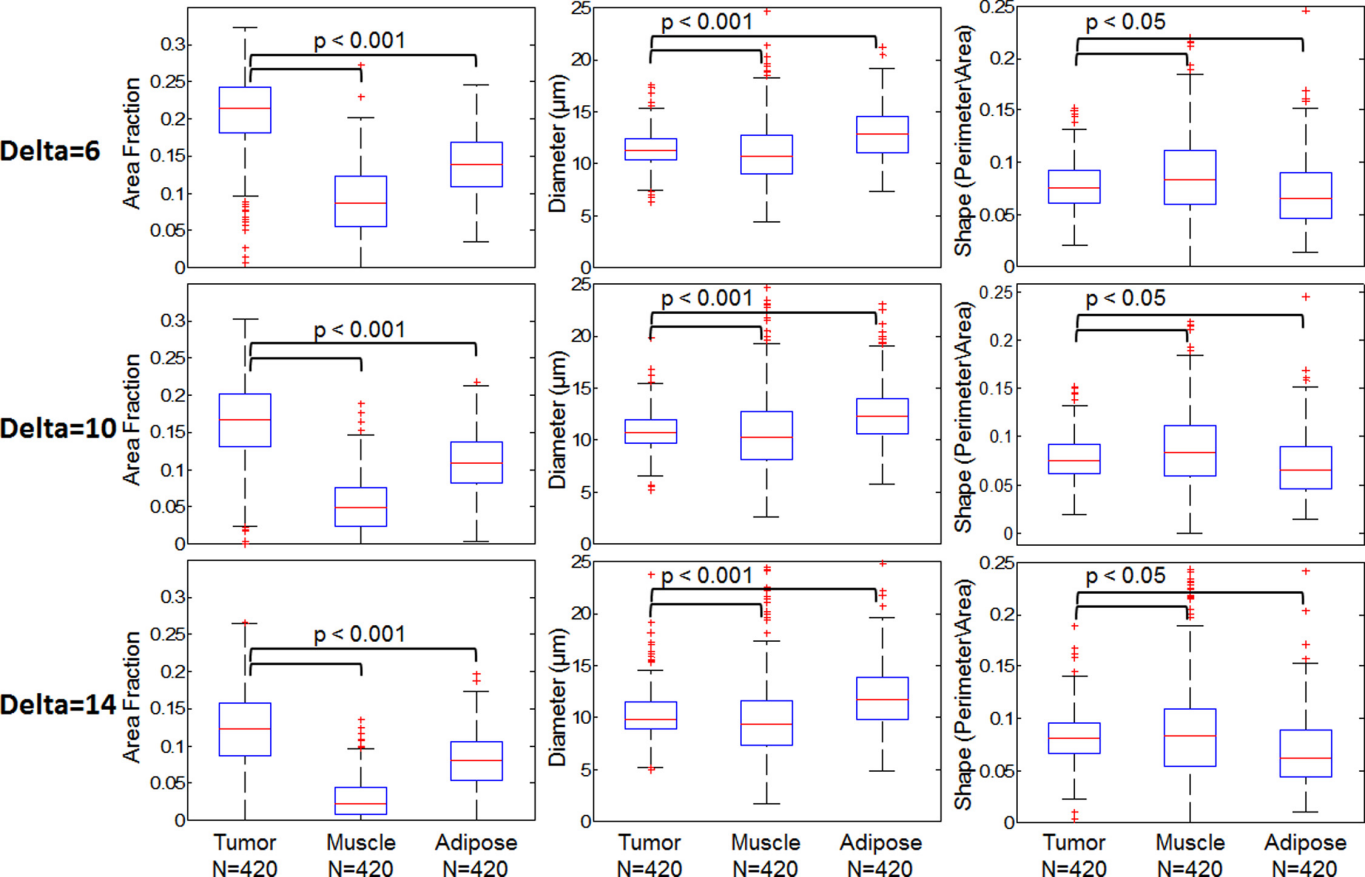
Distribution of calculated variables used for logistic regression model. Box plots of the three variables, area fraction, diameter, and shape for three different delta values. These include all 50x50 pixel bins from the training dataset, N = 420 tumor bins and N = 840 normal bins (420 muscle and 420 adipose). A Wilcoxon rank-sum test was used to compare the distributions and calculate the corresponding p-value. Based on the results, the tumor and normal distributions were statistically different in all three variables, so they were included as inputs into the logistic regression model.

Following the procedure in the methods section, the training dataset of 30 tissue specific images was subdivided into N = 1260 50x50 pixel bins. The area fraction, average diameter, and average shape were calculated for all bins based on the MSER segmentation output for the three delta values. These calculated values of all bins from the training dataset are displayed as boxplots and shown in Fig 7.

**Fig 8:**
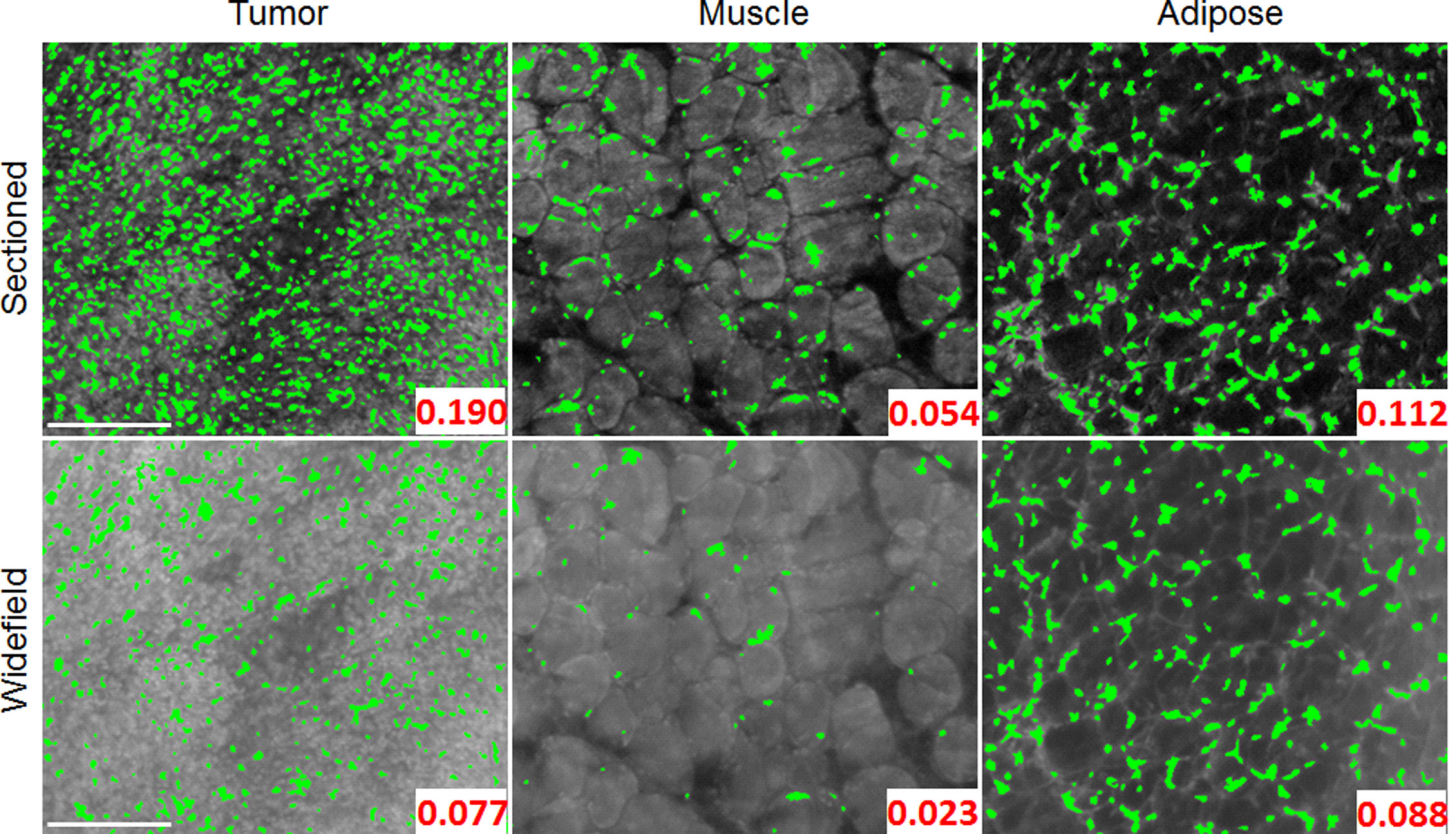
Segmented tissue images using the finalized MSER parameters. Application of the finalized set of MSER parameters to both the uniform and sectioned representative image set. It is evident that the sectioned images provide a clear benefit with contrast enhancement which assists the MSER algorithm in accurately identifying nuclei. The finalized set of MSER parameters used for these segmentation results were MinArea = 3, MaxArea = 15, MinDiversity = 0.5, MaxVariation = 2.5, and Delta = 10. The segmented area fraction is displayed in the bottom right of each image. It is apparent that a lower segmented area is seen in widefield illumination images, due to the decreased contrast. Scale bars are 200 μm.

Fig 7, shows the calculated set of variables for MSER parameter Delta = 6, 10, and 14. In the case of area fraction (left column of boxplots), it is expected that a higher number of nuclei would be present in tumor tissue vs. normal tissue. This is quantitatively supported by the data and changes in Delta do not alter this trend. Furthermore, the trends observed for average diameter and shape are not significantly different among the three Delta settings tested. Therefore, based on these quantitative results and the previously shown qualitative results (in Fig 6), it was determined Delta = 10 was an appropriate setting for this parameter in all subsequent MSER analysis. The three previously shown representative images of tumor, muscle, and adipose are shown again in Fig 8 with the finalized MSER parameter set. Both the sectioned and widefield images are included to demonstrate the importance of contrast enhancement using SIM.

Once the setting for Delta was finalized, the data from the 50x50 pixel bins were input to the logistic regression to estimate coefficients for β_0_, β_1_, β_2_, β_3_. First, the classification model was constructed using all three of input variables. Three additional classification models were constructed using two input variables, where one variable was left out of each iteration. The estimated logistic regression coefficients of each model are shown in Table 2.

**Table 2:** Logistic Regression coefficients.

	Variables Used in Logistic Regression			
	Area Fraction	Area Fraction	Area Fraction	-
	Average Diameter	Average Diameter	-	Average Diameter
	Average Shape	-	Average Shape	Average Shape
β_0_	2.659	1.374	-4.460	1.3587
β_1_	44.074	43.818	30.12	N/A
β_2_	-0.962	-0.870	N/A	-0.2114
β_3_	-0.8652	N/A	12.09	-5.1622

Model coefficients calculated using logistic regression for each combination of the three input variables.

**Fig 9:**
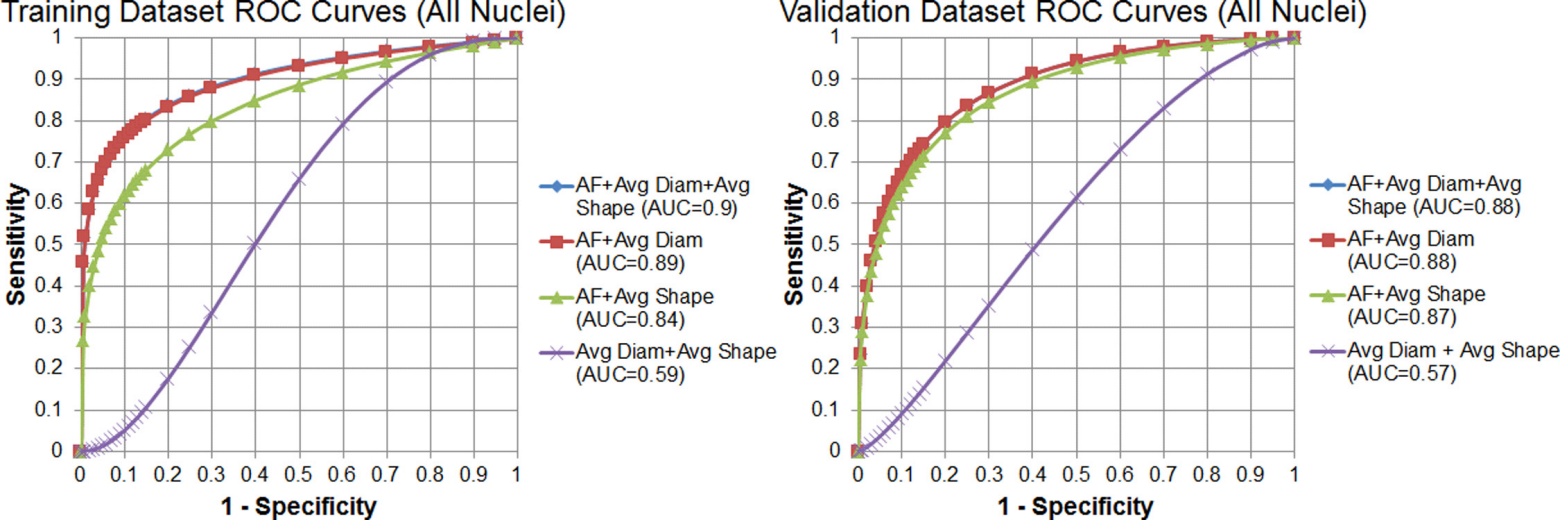
ROC curves demonstrating the accuracy of the logistic regression model. The receiver operating characteristic (ROC) curves (top-left) were generated when logistic regression model was applied to the original training dataset of 30 site specific ROIs: 10 tumor sites vs. 20 normal sites (10 muscle and 10 adipose). The specific combinations of variables used as input to the logistic regression model are shown in the legend. The validation dataset contained 25 additional images (10 tumor, 10 muscle, 5 adipose) that were separate from the training dataset.

Using these estimated coefficients, the predictor value *p*_*i*_ was calculated for each bin. This predictor value is used as the binary classification parameter where α is a threshold and the bin is declared tumor if *p*_*i*_ > α. A receiver operating characteristic (ROC) curve was generated by varying α and is shown in Fig 9. For comparison, three other ROC curves are also shown that were generated from a logistic regression models built using only two of three variables. In addition, the area under the curve (AUC) for each ROC curve was calculated and is displayed in the legend. The AUC provides a measure for the performance of each model. The results are shown for both the training and validation sets.

As shown by the ROC curve, the model constructed using all three variables performed exceptionally well (AUC = 0.9). To test the unbiased accuracy of the site level model, a separate validation dataset was selected. This validation dataset contained 25 additional images (10 tumor, 10 muscle, 5 adipose) that were separate from the training dataset. Only 5 adipose images were selected for this validation dataset due to the smaller number of adipose sites found in the original dataset of 23 margins. As with the training dataset, each image in the validation dataset was divided into the 50x50 pixel bins and the model predictor value *p*_*i*_ was calculated for each bin. These ROC curves demonstrate that the model constructed with only two-variables (area fraction and average diameter) performs almost identically compared to the three-variable model (area fraction, average diameter, and average shape). Based on the ROC curves, the model was able to classify tumor and normal tissue with 77% sensitivity and 81% specificity (Youden’s index). For an unbiased measure of the model performance, it was applied to a separate validation dataset that resulted in 73% sensitivity and 80% specificity.

### Application to full margin images

**Fig 10:**
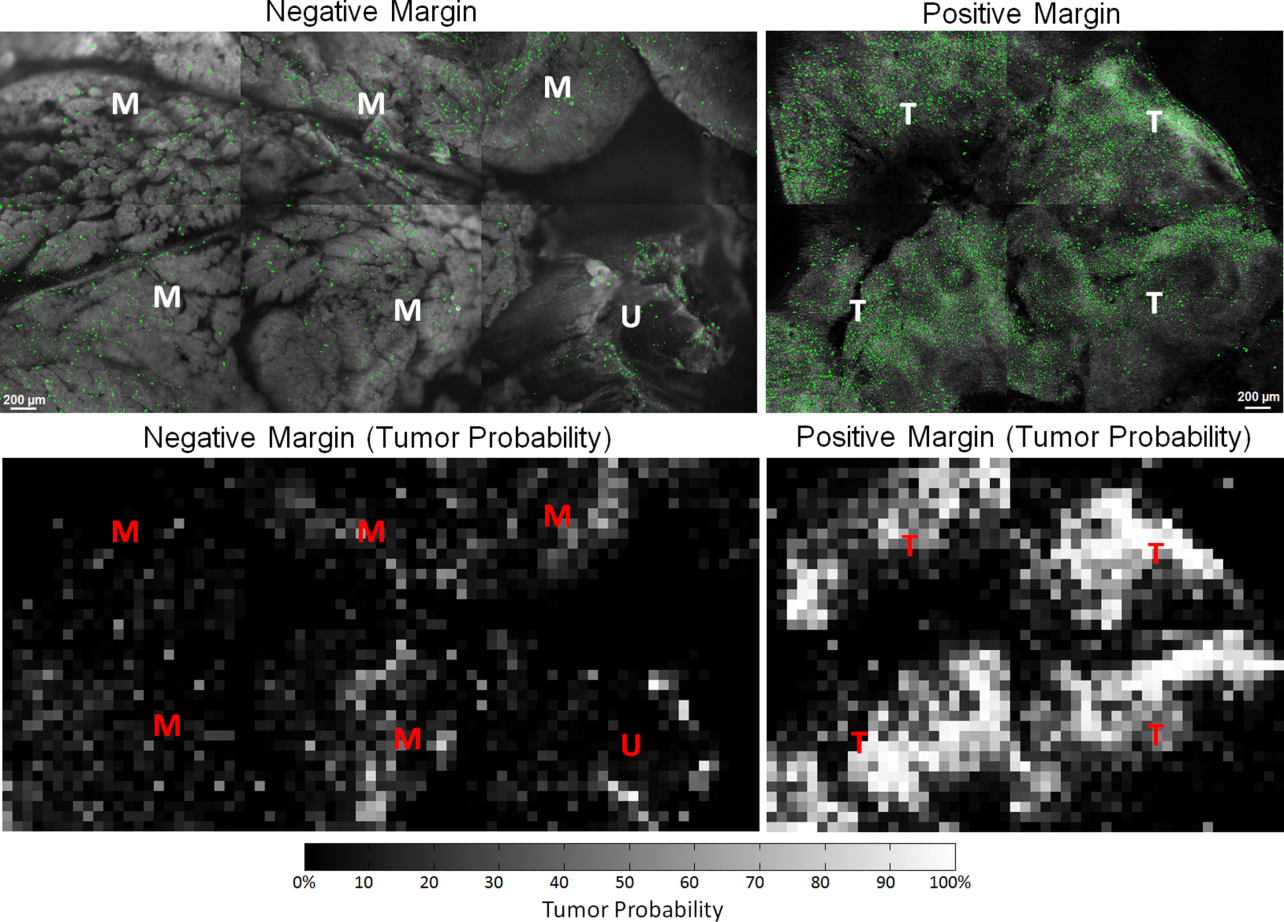
Full margin images acquired with the SIM system. Representative images of a positive margin and negative margin diagnosed through H&E sections. These SIM images shown here have also been analyzed with MSER and the segmented regions are highlighted in green. In addition, specific tissue types were identified by the pathologist in the SIM images and labeled with T (tumor), M (muscle), A (adipose), and U (unknown). The corresponding grid analysis and tumor probability are also displayed as heatmaps.

The final step of the analysis was to demonstrate the feasibility of applying the site level predictive model to three representative positive and negative full margin images. The margin size from each mouse varied based on the tumor size, but most margins were covered using 4 or 6 separate images. The typical image configuration of the margins was either 2x2 or 2x3. In order to fully visualize each margin, multi-image mosaics were generated by placing each image site in its proper physical location, while also accounting for the overlap among adjacent images. An examples of one positive and one negative margins is shown in Fig 10. The classification of an area as positive or negative was provided by the pathologist using the post-operative H&E sections. In addition, the pathologist also inspected the SIM images of these margins, while blinded to her original H&E diagnoses. Using the SIM images alone based on similarity of appearance to H&E, she identified specific tissue types that are also labeled in Fig 10 as T (tumor), M (muscle), A (adipose), or U (unknown).

Following the procedure of the site level images, MSER was then applied to these six full margin images to segment features. The segmentation results of the sectioned images are shown in with the MSER-segmented regions false-colored in green. The tumor probability is represented as a heat map for each margin, which was calculated from the predictive model. From this visualization, it is clear that a larger portion of bins in the positive margins correspond to a high tumor probability. This is an expected result since residual tumor remaining on the positive margin would cause many of the bins to display a high probability. From a quantitative standpoint, less than 1.2% of bins were assigned a >50% tumor probability in the negative margin. In contrast, over 14.8% had a calculated tumor probability of >50% in the positive margin.

### Presence of other tissue types

**Fig 11:**
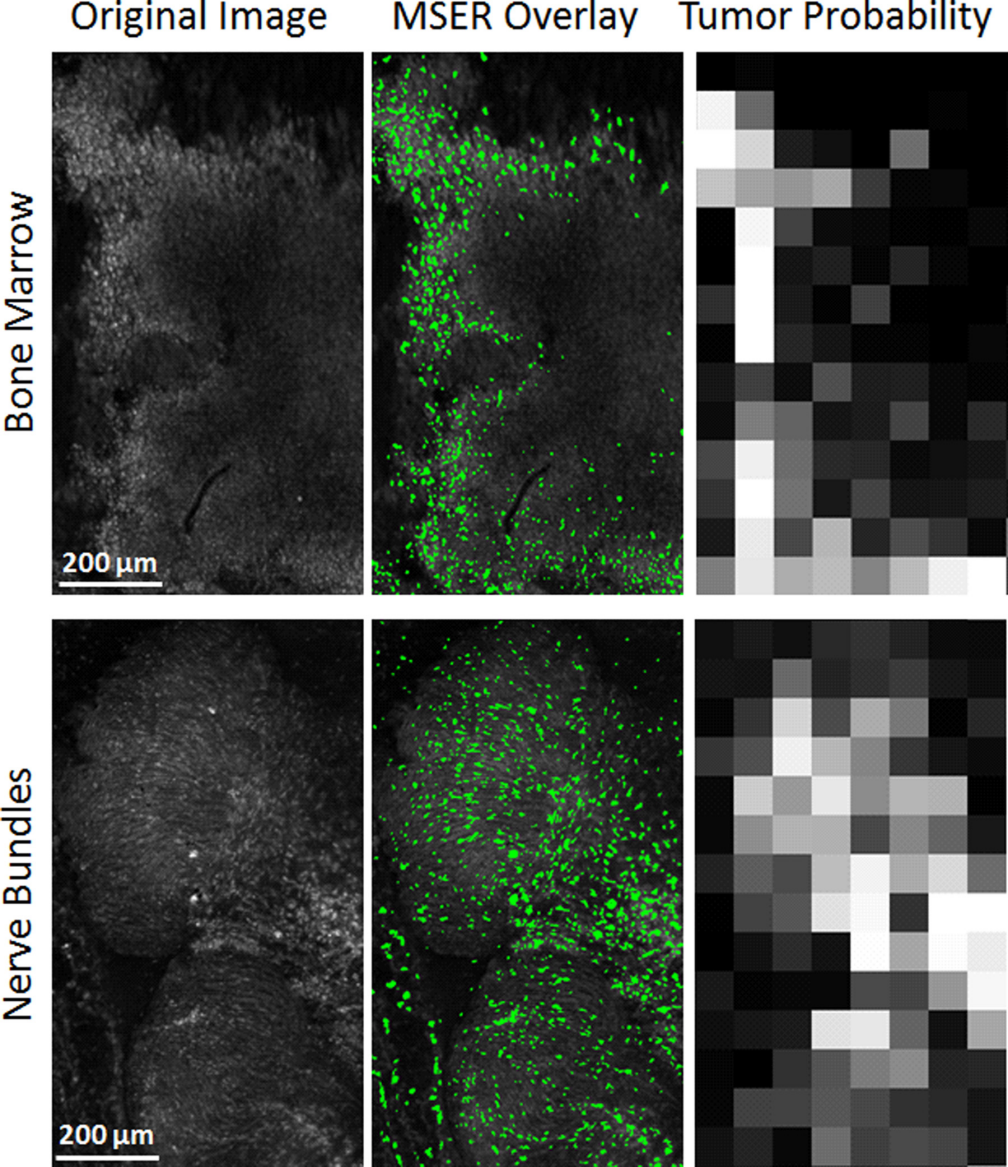
Other tissue types found in margin images. Examples of bone marrow and nerve bundles taken from our dataset. The corresponding MSER segmentation analysis and tumor probability heatmap are also shown. It should be noted that these are two benign tissue types, but due to their high cellular and fibrous composition, the classification algorithm incorrectly identifies these tissue types as areas of tumor.

Once the site-level grid analysis method was applied to this representative set of whole margins, the tumor probability heat map provided a visual representation to match the automated diagnosis model with the diagnosis by the pathologist. The tissue regions labeled by the pathologist as tumor correspond to a high tumor probability, while muscle and adipose regions displayed a low tumor probability. While it was true that tumor, muscle, and adipose tissue are the most common occurring tissue types, a closer inspection of whole margins indicated the presence of a number of additional tissue types. Some examples include fascia, skin, lymph nodes, nerve bundles, and bone marrow, which are all benign tissue types, but are also dense with cell nuclei. The pathologist identified these other tissue types on the H&E sections and also through inspection of the SIM images. Two of these additional tissue types, nerve bundles and bone marrow are shown in Fig 11. From the results of the 50x50 pixel bin grid analysis, these tissue types are assigned with a high tumor probability value and could further contribute to false positives in our data set. In our dataset, these additional tissue types appeared far less frequently than tumor, muscle, and adipose. Due to this limitation, there was an insufficient number of representative images for these additional benign tissues to include them in the training dataset for the site level tissue classification model.

## Discussion

Pathologists heavily base their classification decision on qualitative visual analysis of histologic patterns based on tissue types and nuclear size, shape, and density in tissue sections. Similarly, in order for quantitative microscopy to be clinically viable, it would not only need the ability to visualize, but also segment nuclei, preferably through an automated algorithm. Visual inspection of SIM images acquired on AO-stained tissue clearly revealed that prominent features appeared as small local intensity maxima. When combined with MSER, these features could be automatically segmented and incorporated into a classification algorithm for the detection of positive margins.

Due to the relatively large per frame field of view (>3 mm^2^), a typical SIM image from the sarcoma margin would commonly contain more than a single type of tissue. Given the variety of tissue types and the respective differences in staining intensity, a single global intensity threshold would be unable to properly segment nuclei over highly varying background intensities. MSER is a robust solution that allows the intensity threshold to be selected locally, which is ideal due to the wide range of nuclear intensities relative to the background. During the process of optimizing the three parameters used in MSER (Delta, MaxVariation, and MinDiversity), area fraction was the initial variable used to gauge the segmentation performance as it was a comprehensive summary variable which encompassed segmented density and size. The example MSER segmented images shown in Fig 8, clearly demonstrates the ability of the algorithm to highlight nuclei among tumor, muscle, and adipose tissues. As expected, the area fraction of segmented nuclei was higher in the tumor image by a factor of 3.51 over the muscle image and 1.70 over the adipose image.

Once it was demonstrated that nuclei were automatically segmented using the MSER algorithm, we focused on developing a predictive model to distinguish and classify different tissue types, using “pure” tissue types for characterization and model construction. Area fraction, average diameter, and average shape were selected as parameters since they were quantifiable metrics of pleomorphic changes that pathologists use to identify tumor tissue sections stained with H&E. Based on the ROC curves, the model was able to classify tumor and normal tissue with 77% sensitivity and 81% specificity (Youden’s index). For an unbiased measure of the model performance, it was applied to a separate validation dataset that resulted in 73% sensitivity and 80% specificity.

In previous work, our group has demonstrated that excluding larger regions segmented during the analysis process yielded improved diagnostic accuracy [36]. A size threshold was also applied to this analysis to generate two additional models: one which included only segmented areas less than 10 μm diameter and another with only segmented areas greater than 10 μm diameter. The ROC curves and AUC of the size thresholded images did not show significant differences from those calculated using all sizes. It should be noted that the imaging system, lateral resolution, FOV, and segmentation algorithm all differ from those used in the previous study.

To verify segmentation accuracy, a phantom study was performed where fluorescent beads with diameters of 10, 6, and 1 um were imaged with the system. After applying MSER to segment and quantify region sizes, 10 and 6 μm diameter beads were accurately segmented and sized (estimated average diameter were 6.30±0.21 and 11.79±1.00 μm, respectively). However, the diameters of 1 μm beads, which are significantly lower than the resolution limit of our system, were severely over-estimated (estimated average diameter was 8.83±0.40 μm). This is a potential source of error as any sub-resolution features imaged with our system would be incorrectly identified as a larger region. The result would be an artificially high area fraction and average diameter, both of which are characteristic of tumor tissue. This likely contributes to the false positive rate of the predictive model that we developed.

Through this work, it has been clearly demonstrated that AO is a useful contrast agent for visualizing specific features in sarcoma margins. Its high fluorescence yield generates more than enough signal for the SIM system to detect. An additional feature of AO that has not been explored is its spectral-dependent features. AO is known to be a metachromatic dye—in monomeric form its fluorescence emission peak is 525 nm, the peak is shifted to 590–630 nm. It has been reported that AO tends to aggregate between myofibrils in skeletal muscle [37]. Thus, imaging the two emission peaks of AO could aid in identifying specifically where muscle is located within an image further improving specificity of this technique.

This work has demonstrated that SIM imaging combined with MSER nuclei segmentation is a powerful tool for distinguishing tumor from muscle and adipose tissue. Staining tissue with AO was a straightforward procedure that required only topical application and no specialized tissue or contrast agent preparation. This allowed the resected tumor to be rapidly imaged after excision (<15 min), a critical requirement for potential intraoperative use. Finally, we have laid the groundwork for future studies and potential modifications to improve the specificity of the system.
